# Experiences of using portable oxygen concentrators delivering oxygen or air among people with fibrotic interstitial lung disease in Australia: a qualitative study

**DOI:** 10.1136/bmjopen-2026-117577

**Published:** 2026-09-21

**Authors:** Mariana Hoffman, Gabriella Tikellis, Joanna Y T Lee, Ching Shan Wan, Yet Khor, Nicole SL Goh, Ian N Glaspole, Jyotika D Prasad, Natasha Smallwood, Christine F McDonald, Anne E Holland

**Affiliations:** 1 Respiratory Research@Alfred, School of Translational Medicine, Monash University, Melbourne, Victoria, Australia; 2 Department of Physiotherapy, Alfred Health, Melbourne, Victoria, Australia; 3 Institute for Breathing and Sleep, Melbourne, Victoria, Australia; 4 NHMRC Centre of Research Excellence in Pulmonary Fibrosis, Sidney, New South Wales, Australia; 5 Department of Respiratory and Sleep Medicine, Austin Health, Melbourne, Victoria, Australia; 6 The University of Melbourne Faculty of Medicine, Dentistry and Health Sciences, Melbourne, Victoria, Australia; 7 Respiratory Medicine, Alfred Health, Melbourne, Victoria, Australia; 8 Respiratory Medicine, The Royal Melbourne Hospital, Melbourne, Victoria, Australia

**Keywords:** Lung Diseases, Interstitial, Oxygen Saturation, QUALITATIVE RESEARCH, RESPIRATORY MEDICINE (see Thoracic Medicine)

## Abstract

**Abstract:**

**Objectives:**

Ambulatory oxygen therapy (AOT), defined as the use of oxygen during activities of daily living and exercise, may be prescribed to optimise quality of life and physical activity in people with fibrotic interstitial lung disease (fILD) with exertional hypoxaemia. The aim of this study was to compare patient experiences of ambulatory therapy with oxygen or air delivered via an identical portable oxygen concentrator (POC) in order to understand perceptions of therapy in both active and sham conditions.

**Design:**

A qualitative study using semistructured interviews with open-ended questions was conducted over the telephone.

**Setting:**

Participants data were used from a 6-month clinical trial of AOT (NCT03737409) in three hospitals in Australia.

**Participants:**

Included participants were randomised 1:1 to receive AOT using a POC (oxygen group) or sham therapy using an identical POC delivering air (air group). Participants were instructed to use the POC whenever they were physically active over 6 months. Participants were blind to group allocation. Interviews were conducted after the intervention period and were recorded and transcribed verbatim, and thematic analysis was used to report on experiences.

**Results:**

23 participants with fILD agreed to be interviewed (12 oxygen group, 11 air group). Participants presented with moderate to severe fILD. Four major themes with subthemes were identified in both groups: benefits of POC use, negative aspects of POC use, equipment features, personal decisions about POC use. Themes and experiences did not differ between oxygen and air groups.

**Conclusions:**

POC use provides benefits and challenges. Shared decision-making and consideration of the balance between perceived benefits and harms are required when prescribing this therapy.

**Trial registration number:**

NCT03737409.

**STRENGTHS AND LIMITATIONS OF THIS STUDY**

- This study used purposive sampling and inductive, reflexive thematic analysis, enabling in-depth examination of participants’ lived experiences.
- Interviews were conducted by experienced qualitative researchers who were blinded to trial group allocation and with no relationship with participants.
- Interviews were conducted by telephone, which could have limited the ability to observe non-verbal cues.
- Recruitment from three metropolitan Australian sites and inclusion of participants with moderate to severe fibrotic ILD may limit transferability.
- Although the sample size was appropriate for the in-depth qualitative approach and included participants from both treatment groups, the experiences of these 23 participants may not reflect those of all people with fibrotic interstitial lung disease or users of other oxygen-delivery systems.

## Background

 Fibrotic interstitial lung diseases (fILDs) are characterised by various degrees of lung parenchymal inflammation and fibrosis. A large percentage of patients develop progressive impairment of gas exchange and lung volumes that can lead to hypoxaemia.^1^ Exertional hypoxaemia is known to be associated with poor prognosis and increased mortality.^2^ More than half of all people with fILD may experience exertional hypoxaemia.^3^

Ambulatory oxygen therapy (AOT), defined as the use of oxygen during activities of daily living and exercise, has been studied as a potential tool to optimise oxygen saturation on exertion and improve functional status and symptoms.^4^ The therapeutic role of AOT in people with fILD remains controversial since most of the studies only demonstrate potential benefits during standardised laboratory assessments, which do not reflect its use in daily living.^5^

AOT is commonly delivered via compressed oxygen cylinders; however, adherence is often poor due to a lack of perceived efficacy and poor portability.^6 7^ Portable oxygen concentrators (POCs) provide a potential alternative oxygen delivery method. A recent randomised crossover trial has demonstrated no differences between nadir desaturation or distance achieved during a 6-minute walking test (6MWT) by patients with fILD using a POC or an oxygen cylinder.^8^ Although almost all participants (80%) in the trial preferred using the POC due to its physical characteristics,^8^ there is a lack of evidence regarding the therapeutic value of AOT delivered using a POC.

Our recent randomised parallel-group trial (PFOX) was the first to evaluate the effects of 6 months of AOT versus air delivered via POC to people with fILD.^9^ The trial found no overall benefit of AOT compared with ambulatory air for physical activity measured by steps per day, although varying responses were observed for health-related quality of life, anxiety, depression, and dyspnoea levels with some outcomes favouring the air group. The study reported a low utilisation of the POC, with both groups averaging only 47 min per day.^9^ Adherence to AOT can be impacted by the patients’ perceptions, feelings and beliefs about the therapy and its impact on their lives.

This study aimed to compare the experiences of people with fILD who participated in the PFOX trial and were randomised to use a POC delivering either oxygen or air, to better understand perceptions of device use.

## Methods

### Study design

This qualitative study used a purposive sample of participants with fILD enrolled in an AOT trial between July 2021 and July 2023. No formal sample size calculation was performed, and data collection continued until thematic saturation was reached.^10^

### Study population

The PFOX trial was a double-blind randomised controlled superiority trial in people with fILD who had isolated exertional hypoxaemia (ClinicalTrials.gov NCT03737409). Participants were randomised 1:1 to receive either ambulatory oxygen via a POC or ambulatory air via an identical sham device for 6 months.^4^ The parent trial assessed physical activity, dyspnoea, health-related quality of life and functional exercise. The quantitative trial results have been reported previously.^9^

Participants were recruited from three trial sites in Melbourne, Australia: Alfred Hospital (n=12), Austin Hospital (n=8) and Royal Melbourne Hospital (n=3), in accordance with the previously described inclusion criteria.^4^ Briefly, eligible participants were aged ≥18 years, had a physician-confirmed diagnosis of fILD, were on stable pharmacotherapy for at least 3 months and demonstrated exertional desaturation (oxygen saturation, SpO_2_≤88% for ≥10 consecutive seconds) during a 6MWT on room air. Exclusion criteria included current use of long-term oxygen therapy defined as Partial pressure of oxygen (PaO_2_)≤55 mm Hg at rest on room air, or PaO_2_ 56–59 mm Hg with evidence of right heart failure, current smoking, pregnancy, current participation in pulmonary rehabilitation, non-ambulant status, hospital admission within the past 30 days or anticipated death or transplant within the study period.

### PFOX trial intervention

During their 6-month participation in the PFOX trial, all participants were encouraged to use the POC (Inogen One G3 HF) at all times when they were moving about, including walking at home or in the community, during exercise or during other activities. Written and verbal education was provided and participants received monthly telephone calls from a blinded investigator to encourage POC use. Participants were provided with standard nasal cannulae and a standard POC carry bag, worn over one shoulder, but had the option to use a backpack if required or available. The POC provided pulse-dose delivery and was used at its maximal flow setting (setting 5). The hours of POC use were recorded automatically by the device throughout the intervention period. Participants were not provided with a pulse oximeter and were not encouraged to monitor oxyhaemoglobin saturation at home.

### Data collection

Interview-based methods were used to gain insight into the participants’ experiences and attitudes capturing diverse and nuanced perspectives. Semistructured interviews with open-ended questions were conducted once, by telephone, with consenting participants who were alone at the time of the interview and in a location of their choice. All participants were asked to reflect on their experiences when using the POC during the trial period. A series of 10 open-ended questions with prompts was developed by the researchers involved in the clinical trial (table 1).

**Table 1:** Interview questions

Interview questions	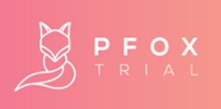
Question 1	Can you tell me about your experience of using your portable concentrator during the trial? Tell me about how you used the portable concentrator outside your home/inside your home/types of activities.
Question 2	How long did you generally use it for? How long, on average per day, have you used the portable oxygen concentrator?
Question 3	Can you tell me about any challenges you experienced using your portable concentrator?
Question 4	Can you tell me about any benefits that you experienced using your portable concentrator?
Question 5	Before you started using the portable concentrator, what did you expect you would experience from it? Can you tell me about how the experience of using your concentrator matched (or didn’t match) your expectations?
Question 6	The aim of using the portable oxygen concentrator was to help you be more active, with fewer symptoms. What do you think about that aim now? How did it make you feel when you used it? How did you feel when you did not use it?
Question 7	How do you find using a portable concentrator? Tell me what you like and dislike about the portable concentrator
Question 8	Is there anything you would change about the portable concentrator to make it easier for people with PF to use in the future?
Question 9	What information or support do you think will be helpful with using a portable concentrator?
Question 10	What advice would you give other people with PF in a similar situation to yourself, who were considering using a portable concentrator?

PF, pulmonary fibrosis.

Interviews were conducted by two female researchers blinded to group allocation and not involved in the PFOX trial and with no relationship with the participants but with experience in qualitative methods and with people with chronic lung conditions (PhD GT, PhD JL). Participants were informed about GT’s and JL’s roles as researchers and the purpose of the research. All interviews were audio-recorded using a professional digital recorder. Interviews were iterative, with follow‐up questions used to confirm or explore participants’ responses where needed. Recordings were transcribed verbatim using a transcription service (Copyright Digital Transcripts, Australia). Participants were offered access to the transcripts via mail or email.

### Data analysis

Inductive, reflexive thematic analysis was conducted independently by two researchers (MH, CSW), guided by the interview guide and following the approach described by Braun and Clarke^11^ and Nowell *et al*.^12^ This analysis followed six steps:

1. Familiarisation with the data through repeated reading of transcripts.
2. Independent inductive coding of meaningful data segments without using a predefined framework by two researchers (MH and CSW).
3. Independent revision and grouping of codes into initial themes by two researchers (MH and CSW).
4. Final revision of transcripts and definition of themes and subthemes through discussion and consensus after data interpretation with participation of a third researcher (AEH) if needed to ensure coherence and representativeness.
5. Definition and naming of themes and selection of illustrative quotes.
6. Production of the results report.

A coding tree was developed on agreement between the researchers on themes and subthemes.^11^ Data management was performed using Excel spreadsheets containing representative quotes organised by participant IDs. These spreadsheets were used collaboratively by the two researchers (MH and CSW) conducting the analysis. Researchers were blinded to group allocation during qualitative analysis; unblinding did not occur until analysis of the PFOX trial was completed and thematic analysis was finalised.^9^ Representative quotes are presented for each theme by participant group.

Results are reported in accordance with the Consolidated Criteria for Reporting Qualitative Research (COREQ) checklist and in accordance with the Standards for Reporting Qualitative Research (SRQR) guidelines (online supplemental files 1 and 2).^13 14^

### Patient and public involvement

People with fILD were involved in the design of the parent PFOX trial, including the selection of the POC used in the study.

## Results

Of the 116 participants enrolled in the parent trial, 23 completed their 6-month follow-up at one of the three Melbourne sites during the qualitative recruitment period and agreed to participate. Interviews were conducted between October 2022 and January 2024. The average interview duration was 20 min and ranged from 15 up to 36 min. POC use was low and varied considerably between participants included in this study with an overall mean use of 0.59 hours per day. At 6 months, mean daily use was 0.62 hours/day in the oxygen group and 0.57 hours/day in the air group.^9^ Twelve participants were allocated to the oxygen group and 11 to the air group. All participants had a confirmed diagnosis of fILD and low levels of physical activity determined by the lower daily step count at the time of trial enrolment (table 2). Transcripts were emailed to three participants who requested them.

**Table 2:** Participant characteristics at baseline

Variable	Mean (SD)
Gender (n) (M/F)	17/6
FVC (% predicted)	71 (17)
TLCO (%predicted)	44.7 (11.2)
6MWT (distance in metres)	432 (106)
Steps per day (n)	3564 (1701)
Resting SpO_2_ (%)	95 (2)
Diagnosis	N
IPF	10
Connective tissue disease-associated ILD	7
Unclassifiable ILD	3
Combined pulmonary fibrosis and emphysema	1
Hypersensitivity pneumonitis	1
Graft vs host disease in progressive fibrotic ILD	1

.FVC, forced vital capacity; ILD, interstitial lung disease; IPF, idiopathic pulmonary fibrosis; 6MWT, 6-minute walking test; SpO2, oxygen saturation; TLCO, transfer factor for carbon monoxide.

**Figure 1:**
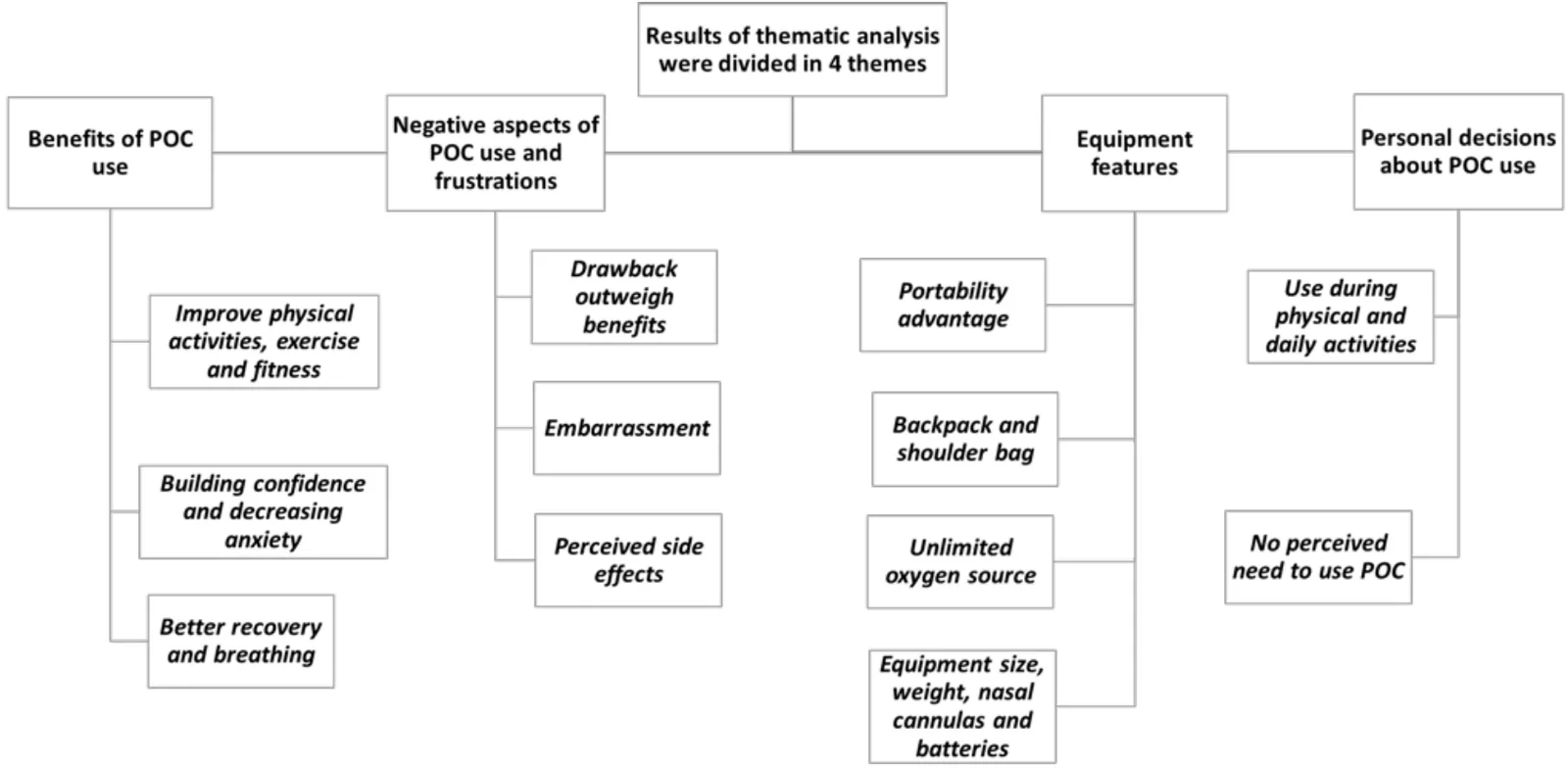
Coding tree showing the four themes and associated subthemes derived from the thematic analysis of participants’ experiences of portable oxygen concentrator (POC) use.

Four major themes were identified across both groups: benefits of POC use, negative aspects of AOT and frustrations, equipment features and personal decisions about POC use (figure 1).

### Theme 1: benefits of POC use

Many participants in both groups reported experiencing benefits from using the POC during the 6-month study period, regardless of group allocation.

#### Improve physical activities, exercise and fitness

Participants in the oxygen group described being able to walk longer, exercise more frequently and push themselves beyond their usual limits when using the POC.

It helped me a lot. In fact, instead of an hour and a half I was doing two hours. So that was walking every day, even seven days a week. […] I found it a lot easier with oxygen to breathe easier and do more walks. I could do it for another half an hour, and without that, there was no way. (P20, O_2_ group)

If I not using it for oxygen I feel if I walk little bit or doing exercise, I’m puff. If I use the oxygen machine, my oxygen machine, you feel ok. (P5, O_2_ group)

Participants in the air group (n=5) also described perceived improvements in endurance and exercise tolerance, suggesting that the POC may have supported improved physical activity:

So, it certainly made an improvement in my ability to do things without being puffed out all the time. (P12, air group)

I really did feel it was helping with the exercise. I think it did help my fitness in one way or another. (P1, air group)

#### Better recovery and breathing

Participants from both groups frequently reported being less breathless, quicker recovery and improved management of physical exertion.

Well, I could breathe easier. It helped me to catch my breath. (P23, O_2_ group)

I didn’t lose the breath very much even when I used to talk, I could talk a little longer. (P3, O_2_ group)

I find that it actually assisted me to recover quicker. If I did something stressful, I recovered it much quicker. (P15, air group)

These accounts highlight that both oxygen and air users experienced perceived improvements in breathlessness and recovery, which may have encouraged greater participation in daily activities.

#### Building confidence and decreasing anxiety

Some participants also reported psychological benefits, including greater confidence and reduced anxiety when engaging in physical or social activities. For some, the POC provided reassurance and a sense of support:

It gave me a bit of a mindset around doing things more often than I would normally have done. It’s given me a bit of confidence to move around a bit more. (P6, O_2_ group)

The confidence and the comfort level that you’d have if you have one. Once you leave the house you can take it with you. (P11, air group)

Overall, these reflections suggest that beyond physiological benefits, the POC had a broader impact on participants’ confidence, motivation and willingness to stay active regardless of group allocation.

### Theme 2: negative aspects of POC use and frustrations

While most participants reported benefits from using the POC, a minority described harms, inconveniences or frustrations associated with its use. These negative experiences varied in nature, ranging from physical discomfort to psychosocial challenges or practical limitations and were described by participants in both groups.

#### Perceived side effects

Some participants experienced discomfort from nasal dryness, shoulder or back strain from carrying the device:

Initially it hurt on my shoulder but I rotate from side to side so that was fine. (P6, O_2_group)

I got a very dry nasal passage and that after about half an hour of heavy breathing, I did find that dried my mouth and nose a little bit. (P1, air group)

So, I found the weight really difficult and if I wore it two days in a row my back was really bad. (P18, air group)

#### Embarrassment

The visibility of the device in public caused self-consciousness or stigma for some participants.

People give you some very strange looks not knowing what’s wrong with you. That was noisy but that only making me feel self-conscious. (P6, O_2_ group)

I’m a bit embarrassed walking around with it so I used to put a coat over it. I just found it a little bit I suppose embarrassing that you have to walk around with it on. (P10, O_2_ group)

I was a little reluctant to wear it in public, I guess that was just a self-conscious problem that I had. (P1, air group)

#### Drawbacks outweigh benefits

Some participants reported difficulties with the weight of the device and the inconvenience of carrying and wearing it. Limited battery life and the need for frequent charging were also common frustrations.

I found it a bit of a nuisance to carry out and in the car or shops, it does create a lot of background noises like pumping, and it’s very noisy. (P4, O_2_ group)

Because they’re all behind my ears (referring to nasal cannula), when it moves it’s causing me to adjust everything, especially when I’m exercising. I don’t realise that I have dropped my hearing aid. (P9, air group)

You were constantly charging the battery, but I don’t think it really did any benefit to me as such when I was wearing it. I didn’t feel any difference. (P19, O_2_ group)

Several participants from both groups expressed disappointment when the POC did not meet their expectations for improving breathing or exercise tolerance.

I just thought I’d be able to breathe a bit easier but it really didn’t seem to make a hell of a lot of difference. (P7, O_2_ group)

Like it’s hard to know whether I'm getting worse and it’s taking longer to recover. Or I was taking longer to recover and it wasn’t helping. So, I really didn’t feel a benefit from it. (P21, O_2_ group)

It made little or no difference to me. It didn’t affect me very much at all. (P15, air group)

The symptoms that I had beforehand, exertion and stuff like that, up hills, even with the concentrator I had the same problems. (P17, air group)

### Theme 3: equipment features

Participants highlighted both desirable and undesirable features of the POC.

#### Portability advantage

The lightweight and portable design was valued by participants.

Very easy to use, and also to recharge, but the batteries take quite a while to recharge. It was lightweight portable. (P6, O_2_ group)

Small easy to carry everywhere, it was the benefit. (P5, air group)

#### Unlimited oxygen source

One participant who also reported having used an oxygen cylinder before appreciated the unlimited oxygen source generated by the POC, with the device able to operate from a power source and its batteries able to be recharged.

The good thing about the portable is the continuous supply, can be 24 hours as long as you plug in the power source, there’s no limit. But with the cylinder, if I finish 2 litres of that I finish 2 litres of that, I have to wait for new supply. (P4, O_2_ group)

#### Backpack and shoulder bag

Participants described different carrying options for the POC, highlighting how each affected usability during daily activities.

I found the side sling as I said very easy if you’re just going to go out to do something where it didn’t get in the way. But if you’re doing something that is very physical or you needed both hands to do, then the backpack was the way to go. (P8, air group)

It’s quite cumbersome, I think the backpack made it a lot bigger object, with the shoulder strap, it was overall a little bit smaller. (P1, air group)

#### Equipment size, weight, nasal cannulas and batteries

Limitations related to device weight, battery life, nasal cannula length and size were frequently noted by participants in both groups (oxygen group, n=8; air group, n=6).

Oh, the weight of it, it does sometimes feel very heavy. I mean when you initially carry on the shoulder it’s ok, but after 5 or 10 minutes, it’s just the weight put on your shoulder. (P4, O_2_ group).

It was a bit heavy to carry around, when I used it, I had to put it in the shopping trolley to walk around with it. (P7, O_2_ group)

I mean I found the long hose annoying because you get all this hose that you got to sort of deal with. You know a shorter hose would be good in that situation. (P21, O_2_ group)

It was hard to concentrate on what you were doing because you had this constant sort of puff, puff in your nostrils and out of the accumulator on your back. (P12, air group)

Every time you take it out, out of the house, in the car, or shopping, you got limited time to use it, because the battery. (P4, O_2_ group)

I used it two to three hours but the batteries were off, that was the only problem. I had two batteries but they still weren’t enough to last all day so to speak. (P17, air group)

### Theme 4: personal decisions about POC use

Participants described when and how they chose to use the POC, and situations in which they felt it was unnecessary.

#### Use during physical and daily activities

Participants in both groups mainly used the POC for outdoor activities, exercise and tasks requiring higher exertion.

I used also for on the bike, I bought myself an exercise bike and put it in the carport, I found that it can push me a bit further along in the bike. (P10, O_2_ group).

When I was outside cutting the grass, I put it on and start the mowing. (P6, O_2_ group).

I did use it, just very shortly while I was bike riding. (P1, air group)

I probably was doing about 20, 25 minutes walking. Whereas on the treadmill I was sort of expanding a little bit more, maybe 30 minutes, easily 25 anyway. (P16, air group)

I used it when I was outdoors mainly, like walking around, playing golf. I used it as a help and stuff like that, doing the gardening. (P17,air group)

So initially I used it whenever I was doing something a little bit strenuous, like for example, tidying up the garage or cleaning up around the house or going for a walk, and all that sort of stuff. (P12, air group)

#### No perceived need to use POC

Some participants considered the device as unnecessary during routine tasks requiring minimal exertion, particularly when the complexity of using the device outweighed the perceived effort of the task.

I don’t see the necessity of me having one and my health condition. I could still move around from point A to point B, fully mobilise without using the machine at all, I had no need for it. (P4, O_2_ group)

In our house we have lots of steps, split level, and I’m up and down them all day, so I don’t feel that I needed it to just function daily at this stage. (P1, air group)

Overall, participants’ experiences varied. Some participants described a substantial perceived benefit from using the POC, particularly reduced breathlessness, increased confidence and greater ability to undertake activities. In contrast, others perceived little or no benefit and experienced the weight, inconvenience or visibility of the device as a major burden. Most participants described a more mixed experience, balancing perceived benefits against the practical challenges of carrying and managing the device. Strongly positive and negative experiences occurred in both the oxygen and air groups.

## Discussion

This is the first qualitative study to explore experiences of POC use in people with fILD that included individuals using the same device delivering oxygen or air. By incorporating both groups from the PFOX trial,^9^ this study provided unique insights into how patients perceive and experience the use of POC independent of the physiological effects of oxygen. Participants across both groups reported similar experiences with the portable concentrator, describing perceived improvements in exercise tolerance, breathlessness, recovery and overall confidence when being active. Beyond physical benefits, participants also highlighted psychological benefits such as increased confidence and reduced anxiety when leaving home or engaging in social and physical activities. These findings suggest that the device contributed not only to perceived improvements in exercise capacity and recovery but also to greater motivation, confidence and willingness to stay active, regardless of whether participants received oxygen or air. At the same time, participants described practical and social challenges, including the device weight, noise, visibility and disruptions to daily routines. The similarity of experiences between groups suggests that perceptions of benefit and burden were not solely driven by oxygen itself but shaped by multiple factors including sensory experience of additional flow of air delivered by the device and the symbolic and emotional meaning associated with ‘being on oxygen’.

A recent scoping review showed that directing a stream of cool air to the face and nose may relieve breathlessness by stimulating the trigeminal nerve, leading to reduced inspiratory drive and modulation of neuronal activity within brain regions.^15^ Although air delivered through the nasal cannula may not have produced the same facial cooling effect, the additional direct airflow to the nose delivered by the POC influenced participants’ perception of breathlessness, consistent with evidence in other chronic lung diseases showing that facial airflow (eg, from a handheld fan) can relieve breathlessness and anxiety.^16–19^ Using the POC may have increased confidence and willingness to undertake activity, regardless of the gas delivered. Contextual and expectancy effects may therefore have influenced both groups. However, similar experiences should not be interpreted as evidence that oxygen acted only through a placebo effect, as it may also have provided physiological benefit.

Breathlessness has been described within a phenomenological framework in which its perception extends beyond physiological impairment and is influenced by psychological, emotional and social factors such as depression, anxiety and socioeconomic status.^20 21^ Because exertional breathlessness involves cognitive, sensory and emotional dimensions, its experience is inherently subjective. Consequently, experiences with AOT are likely to vary between individuals, with perceived improvements in breathlessness reflecting more than physiological change alone. There are currently no data to suggest that demographic or physiological characteristics can distinguish people who benefit from AOT from those who do not. Understanding the role of AOT and POC therefore requires a phenomenological approach that accounts for participants’ lived experiences and the personal meaning of their symptoms.^22^ In the absence of established predictors of benefit, an individual trial of AOT with review of symptom response, activity goals and device burden may support shared decision-making.

Barriers to POC use identified in this study are consistent with reports across ILD and other chronic lung diseases including inconvenience during outdoor use, social stigma, limited battery life and device weight.^23–25^ Social discomfort and the practical burden of carrying a POC were reported regardless of whether participants were using oxygen or air.^23^ Potential unintended harms extended beyond physical discomfort and psychosocial effects may have discouraged POC use in public. However, the interviews did not assess whether non-use resulted in physiological or clinical harm. Portability and ease of use were viewed as positive features, consistent with prior comparisons of oxygen cylinders and POCs during the 6MWT, with the present findings showing these benefits extend to daily activities.^8^ However, participants’ experiences indicate that further improvements in device design, weight, portability and visibility are needed to better support people using AOT.

The relatively low POC use observed in the parent trial should also be considered when interpreting participants’ experiences.^9^ Limited exposure to the device may have reduced opportunities to experience both potential benefits and burdens of POC use. However, the qualitative findings also provide insight into factors that may themselves have contributed to low use, including the practical limitations of the equipment and participants’ individual decisions about when the perceived benefit justified the burden of using the device.

This study has several strengths including the use of a rigorous blinding strategy: participants, interviewers, and researchers analysing the data were all blinded to study allocation, reducing the risk of bias in reporting and interpretation. The study also used a consistent semistructured interview guide and applied systematic qualitative analysis methods to enhance credibility and trustworthiness. However, there are some limitations. Only a small proportion of the total trial cohort participated in the interviews, which may limit the transferability of findings. Participants of the main trial were likely to be self-selected to be more receptive of the intervention. The interviews did not specifically explore tolerance of pulse-dose delivery, preference for continuous flow or difficulties with breath-triggered delivery. The study was conducted at only three of the six original trial sites that were located in Melbourne, which may reduce representativeness of broader ILD populations. The SpO₂ was not measured during individual episodes of POC use; therefore, perceived benefits could not be directly linked to an objective physiological response.

### Conclusions

This study highlights both the benefits and challenges of POC use in daily life for people with fILD. Ambulatory oxygen and ambulatory air produced similar perceived benefits and burdens, supporting findings from the PFOX trial and underscoring the need for an individualised approach to AOT prescription. Participants’ experiences varied, with some reporting little benefit from oxygen despite exertional desaturation, while others described meaningful physical and psychological improvements with either oxygen or air. These findings emphasise the importance of shared decision making when weighing perceived benefits against potential harms. Ongoing challenges related to device design and practical use indicate opportunities to improve POC technology and support to enhance patient experience and adherence.

## Supplementary material

10.1136/bmjopen-2026-117577 online supplemental file 1

10.1136/bmjopen-2026-117577 online supplemental file 2

## Data Availability

No data are available.
