# Supplementary material for: Experiences of using portable oxygen concentrators delivering oxygen or air among people with fibrotic interstitial lung disease in Australia: a qualitative study
Source: BMJ Open. 2026 Sep 21;16(9):e117577. doi: 10.1136/bmjopen-2026-117577 (PMC13599980; doi:10.1136/bmjopen-2026-117577)
Supplement: Supplementary file 2 [file bmjopen-2026-117577.reviewer_comments.pdf]

PEER REVIEW HISTORY

BMJ Open publishes all reviews undertaken for accepted manuscripts. Reviewers are asked to complete a checklist review form and are provided with free text boxes to elaborate on their assessment. These free text comments are reproduced below.

ARTICLE DETAILS

Title (Provisional)

Experiences of using portable oxygen concentrators delivering oxygen or air among people with fibrotic interstitial lung disease in Australia: a qualitative study

Authors

Hoffman, Mariana; Tikellis, Gabriella; Lee, Joanna Y T; Wan, Ching Shan; Khor, Yet; Goh, Nicole SL; Glaspole , Ian N; D Prasad, Jyotika; Smallwood, Natasha; McDonald, Christine F.; Holland, Anne E

VERSION 1 - REVIEW

**Reviewer**	**1**
**Name**	**Mattila, Tiina**
**Affiliation**	**HUS Helsinki University Hospital, Pulmonary diseases**
**Date**	**16-Mar-2026**
**COI**	**None**

I thank for the possibility to review this article about "Experiences of using portable oxygen concentrators delivering oxygen or air: a qualitative study". This interesting study found that there were no differences in experiencies in daily life between those receiving oxygen and air via POC.

The English and text were clear and easy to read.

Major comments/ general problems

- small population size

- The results are quite limited. There was no standardized QoL or physical capability questionnaire (st George etc) was made. Additionally the end point was limited in study. No such end point as hospitalization, disease progress etc? Or 6mwt before and after the study?

- adherence in oxygen/ portable device? not measured?

Minor comments

Methods

- How was the saturation in rest in study population?

- What was yhe definition for moderate/ severe ILD?

Discussion

- please shorten the chapter one; it should be a short conclusion about your findings in max 5-7 lines; after that in next chapters compare your results to previous results

---

**Reviewer**	**2**
**Name**	**Costello, Richard**
**Affiliation**	**Royal College of Surgeons Ireland, Respiratory Medicine**
**Date**	**12-Apr-2026**
**COI**	**None**

The aim of this well written paper was to look at patient's perspective on the merits and drawbacks of portable oxygen concentrators (POC) . The population was a cohort of people who participated in a relatively utility trial of air vs oxygen delivered by POC. As this is a qualitative study the results really reflect the general themes that were explored in the interactions between the researchers and the patients. Very reasonably from my point of view they used a semi-structured approach. To this end they discussed the pros and cons of oxygen, the technology and the patient's response to these. The patient's responses are important for clinicians to be aware of, as they will provide information for training in shared decision-making conversations that may come up in future situations on the benefits of portable oxygen concentrators for the relief of breathlessness or the maintenance of fitness.

This raises my major question of "effect size" in other words, were there some super responders/negativists in this interview set? In other words were there people who really felt that they perceived a benefit/downside compared to a more general situation where most people recognized the pros and cons and that they just managed. I am asking to give some scale to depth of feeling and in what domain did this occur?

Minor comments

1.Just for clarity I just want to be sure that the patients didn't have an oxygen saturation monitor during the study.

2. Is it possible to tell how many steps were taken in periods of exercise as opposed to brief periods of activity around the house for example going to the bathroom which may be "planned active use" scenarios as opposed to "more trouble than it's worth" scenarios.

3.From the patient statements listed in the paper it seems adherence to the POC was notable when participants were engaged in solo activities such as exercising on a stationary bike or playing golf as opposed to when they were out in public. Is there more data to explore scenarios where people felt benefit which may add another theme into a shared decision conversation.

**Reviewer**	**3**
**Name**	**Sangey, Esmail**
**Affiliation**	**Shree Hindu Mandal Hospital, Critical Care Unit**
**Date**	**11-Jun-2026**
**COI**	**None**

The manuscript is well written which addressed an important clinical question, in regards to compare experiences with oxygen versus sham air delivered by identical POCs.

Main area for improvement: More detailed exploration of why participants receiving air experienced benefits similar to those receiving oxygen, and a fuller discussion of placebo, airflow, and psychological mechanisms underlying perceived benefit. The way it was well described on the discussion part "A recent scoping review showed that directing a stream of cool air to the face and nose may relieve breathlessness by stimulating the trigeminal nerve, leading to reduced inspiratory drive and modulation of neuronal activity within brain regions." Does it mean even Oxygen delivery have more placebo effect to alleviate from anxiety or true breathlessness?

One area that could be explored further is whether use or none-use of the POC (oxygen or air) may have caused unintended harms beyond the practical inconveniences described.

The study makes a valuable contribution to understanding patient experiences with ambulatory oxygen therapy in fibrotic interstitial lung disease and is suitable for publication after minor revision.

**Reviewer**	**4**
**Name**	**Hughes, Michael**
**Affiliation**	**Hampshire Hospitals NHS Foundation Trust**
**Date**	**14-Jun-2026**
**COI**	**None**

Overall, it is a well-structured and logically presented qualitative study showing good real-world data and patient experience. The paper clearly describes the link to the previously published PFOX study and sets out logically the findings in a thematic approach. The examples chosen in the most part illustrate the overall thematic findings and the disucssion place the findings in the wider conversation around the benefits of AOT.

The paper acknowledges the lack of transferability and the study design is open to bias, but allows deeper questioning.

There are some aspects that are still unclear and impact the quality of the analysis:

1. How was adherence monitored/estimated? The percieved benefits may be significantly impacted by the overall adherence to therapy, but there is no comment if this was measured or how.

2. There is no reference to any objective SpO2 improvement with POC O2 delivery (i.e. is the device at least showing a physiological improvement)? There is a potential ceiling effect of POC devices and thus the conclusions and comparisons would be more robust if there was evidence it did have a meaningful physiological impact that does or does not translates to a poor real-world improvement in QoL/confidence/exercise tollerance.

3. Not all patients get on with a pulse-dose delivery style, but seems to be little comment on this aspect.

4.Can authors confirm high-flow canulae were used with the device?

Specific comments on aspects of the manuscript:

Ref 11 in methods seems out of place (p8) as Nowell et al. (2017) does not seem to specifically link to the development of the coding tree and themes. On further review, reference 11 and 12 may be in the incorrect order as page 5 lists " Braun and Clarke (2006) and Nowell et al. (2017). as ref 11 and 12 respectively. Check please.

It is unclear how the 23 invited participants from the 116 enrolled in the main study were selected. On page 6, it is reported that "all participants were invited to take part" but then on page 9 it says of the 116 total, 23 were invited and all consented to take part in the study. Please clarify how the 23 were selected - or make clear that only 23 made it to the 6-month follow-up and thus 23 represents all participants.

Table 2 should also include Interstitial Pulmonary Fibrosis in the legend.

The overall thematic analysis would be strengthened if the total number of participants coded within each section from the O2 and air group were shown as well as the example quotes. i.e. "Participants from both groups frequently reported being less breathless, quicker recovery, and improved management of physical exertion". (Overall O2 n=4; air n=2) (p11). It would allow for a better comparison between the two groups for each theme, rather than just the examples given which can easily be biased from the examples chosen.

In addition, the above point would also benefit when "participants" is used but only one example given - i.e. 3.2 (p14). Clarify the total number of participants supporting this finding.

It is slightly unclear what the comparison in theme 3 is made to - in the study design these patients are AOT neive, so how can the participants notice an advantage of POC over any other method of O2 delivery? (p13-14). Please clarify what prior experiences allow patients to make this comparison, and to what?

p14 section 3.3 use of "air group" rather than just "air" - consistency. Correct any other instances.

The discussion draws on the key findings of the study and contextualises them in the current knowledge base. The authors identify the need of shared decision making because of the mixed benefits percieved (p18), but little insight if any of the data collected helps predict which patients will or will not get a benefit - indeed if there is any evidence to help in this decision. The discussion would be further strengthened if a comment could be made on this aspect.

The reference base is self-citation heavy. Whilst lots of relevent work has been published by this group, it would also be good to acknowledge briefly the wider base (i.e. studies also looking at POC devices and acceptability to patients, and different disease areas). I am also unclear of the difference between reference 9 and 13.

Please note also that the SRQR checklist is not cited within the main article as outlined in section 2 of the checklist (which is correctly completed and included as a separate file). Please follow the guidance in the document.

VERSION 1 - AUTHOR RESPONSE

Reviewer: 1
Dr. Tiina Mattila, HUS Helsinki University Hospital
Comments to the Author:
I thank for the possibility to review this article about “Experiences of using portable oxygen concentrators delivering oxygen or air: a qualitative study”. This interesting study found that there were no differences in experiences in daily life between those receiving oxygen and air via POC.

The English and text were clear and easy to read.

Major comments/ general problems
- small population size

Thank you for raising this point. This was an embedded qualitative study designed to provide an in-depth exploration of participants’ experiences of using a portable oxygen concentrator, rather than to estimate treatment effects or make statistical comparisons between groups. Sample size was therefore guided by the adequacy and extent of the qualitative data rather than by a statistical power calculation. We interviewed 23 participants, including 12 participants allocated to oxygen and 11 allocated to air. Data collection continued until the research team considered that sufficient depth and diversity of experiences had been captured and no substantively new concepts were emerging from the interviews.
We agree that the findings may not represent the experiences of all people with fibrotic interstitial lung disease who use ambulatory oxygen. We have therefore strengthened the limitations section to acknowledge the sample size and the potential limitations to transferability as follows:

> *“Although the sample size was appropriate for the in-depth qualitative approach and included participants from both treatment groups, the experiences of these 23 participants may not reflect those of all people with fibrotic interstitial lung disease or users of other oxygen-delivery systems.”*

- The results are quite limited. There was no standardized QoL or physical capability questionnaire (st George etc) was made. Additionally, the end point was limited in study. No such end point as hospitalization, disease progress etc? Or 6mwt before and after the study?

Thank you for this comment. The purpose of the present study was specifically to explore participants' experiences of using portable oxygen concentrators in daily life using qualitative interviews. It was not designed to evaluate the clinical efficacy of oxygen or compare quantitative outcomes between the oxygen and air groups. Those outcomes have already been reported in the primary PFOX trial publication. (PMID: 41765033) (https://www.thelancet.com/journals/lanres/article/PIIS2213-2600(26)00005-6/fulltext)

We believe this qualitative study provides critical insights which complement the quantitative data reported in the RCT. We have revised the Methods to clarify the distinction between the outcomes of the parent trial and the focus of this embedded qualitative study.
Methods Page 4 as follows:

> *"The parent trial assessed physical activity, dyspnoea, health-related quality of life and functional exercise. The quantitative trial results have been reported previously."*

- adherence in oxygen/ portable device? not measured?

Thank you for highlighting this issue. Portable oxygen concentrator use was recorded automatically by the device throughout the six-month intervention period and has been reported in the primary PFOX trial publication. As participants were encouraged to use the device whenever they were moving about and were not prescribed a minimum duration of daily use, we describe this measure as "device use" rather than "adherence"

We have added the device-use data for the participants included in the qualitative study to the results and clarified how device use was recorded as follows:

> *Results:*
> *Page 5: " The hours of and POC use were recorded automatically by the device throughout the intervention period. Participants were not provided with a pulse oximeter and were not encouraged to monitor oxyhaemoglobin saturation at home."*
>
> *Page 8: " POC use was low and varied considerably between participants included in this study with an overall mean use of 0.59 hours per day. At six months, mean daily use was 0.62 hours/day in the oxygen group and 0.57 hours/day in the air group.[9]*

Minor comments

Methods
- How was the saturation in rest in study population?

Thank you for this comment. The inclusion criteria for the PFOX trial specified that participants had isolated exertional desaturation and were normoxic at rest. This has now been clarified in the methods section (Page 4) as follows:

> *"The PFOX trial was a double-blind randomised controlled superiority trial in people with fILD who had isolated exertional hypoxaemia."*

In addition, we have now included resting oxygen saturation for participants in the qualitative study in the demographic characteristics presented in the Results section (Table 2). Consistent with the inclusion criteria, mean SpO2 at rest was 95%.

- What was the definition for moderate/ severe ILD?

Thank you for raising this point. We agree that the term "moderate/severe ILD" was insufficiently defined, our intention was to describe the degree of physiological impairment in the study population rather than to apply a formal disease-severity classification. As lung-function impairment alone does not provide a comprehensive assessment of disease severity, we have removed the term "moderate-to-severe ILD" throughout the manuscript.

Discussion
- please shorten the chapter one; it should be a short conclusion about your findings in max 5-7 lines; after that in next chapters compare your results to previous results

Thank you for this suggestion. We have shortened the opening of the Discussion to provide a more concise summary of the principal findings.

Reviewer: 2
Prof. Richard Costello, Royal College of Surgeons Ireland
Comments to the Author:
The aim of this well written paper was to look at patient's perspective on the merits and drawbacks of portable oxygen concentrators (POC) . The population was a cohort of people who participated in a relatively utility trial of air vs oxygen delivered by POC. As this is a qualitative study the results really reflect the general themes that were explored in the interactions between the researchers and the patients. Very reasonably from my point of view they used a semi-structured approach. To this end they discussed the pros and cons of oxygen, the technology and the patient's response to these. The patient's responses are important for clinicians to be aware of, as they will provide information for training in shared decision-making conversations that may come up in future situations on the benefits of portable oxygen concentrators for the relief of breathlessness or the maintenance of fitness.

This raises my major question of "effect size" in other words, were there some super responders/negativists in this interview set? In other words were there people who really felt that they perceived a benefit/downside compared to a more general situation where most people recognized the pros and cons and that they just managed. I am asking to give some scale to depth of feeling and in what domain did this occur?

Thank you for this comment. Participants' experiences varied considerably. Some described a strong perceived benefit, particularly in relation to breathlessness, confidence and ability to undertake activities. Others reported little or no perceived benefit and described the weight, inconvenience and visibility of the device as substantial barriers to use. However, the more common experience was mixed: participants recognised potential benefits while simultaneously managing the practical burden of the device. We have revised the Results (Page 15) to make this spectrum of experiences more explicit as follows:

> *"Overall, participants' experiences varied. Some participants described a substantial perceived benefit from using the POC, particularly reduced breathlessness, increased confidence and greater ability to undertake activities. In contrast, others perceived little or no benefit and experienced the weight, inconvenience or visibility of the device as a major burden. Most participants described a more mixed experience, balancing perceived benefits against the practical challenges of carrying and*

> *managing the device. Strongly positive and negative experiences occurred in both the oxygen and air groups.”*

Minor comments
1.Just for clarity I just want to be sure that the patients didn't have an oxygen saturation monitor during the study.

Participants were not provided with a pulse oximeter for continuous monitoring during the six-month intervention. The masking assessment reported in the parent trial supported effective blinding, suggesting that the experiences described in this study were unlikely to have been influenced by participants’ knowledge of their treatment allocation. (PMID: 41765033). We have added to the methods on page 5 as follows:

> *“Participants were not provided with a pulse oximeter and were not encouraged to monitor oxyhaemoglobin saturation at home.”*

2. Is it possible to tell how many steps were taken in periods of exercise as opposed to brief periods of activity around the house for example going to the bathroom which may be “planned active use” scenarios as opposed to “more trouble than it's worth” scenarios.

Thank you for this comment. Unfortunately, it was not possible to determine the number of steps accumulated during specific activities. The primary physical activity outcome in the parent trial was total daily step count, and participants did not complete an activity diary or record the timing and context of individual activities. Therefore, we could not distinguish steps taken during planned exercise from those accumulated during brief or incidental activities around the home.

3.From the patient statements listed in the paper it seems adherence to the POC was notable when participants were engaged in solo activities such as exercising on a stationary bike or playing golf as opposed to when they were out in public. Is there more data to explore scenarios where people felt benefit which may add another theme into a shared decision conversation.

Thank you for this observation. We revisited the coded interview data to examine whether POC use, or perceived benefit differed between solo activities and activities undertaken in public.
Participants described using the POC across several contexts, including exercise at home, walking, household activities, shopping, travel and social outings. However, the interviews did not systematically explore or compare solo versus public activities, and the available data are insufficient to identify a consistent pattern or support an additional theme.

Reviewer: 3
Dr. Esmail Sangey, Shree Hindu Mandal Hospital
Comments to the Author:
The manuscript is well written which addressed an important clinical question, in regards to compare experiences with oxygen versus sham air delivered by identical POCs.
Main area for improvement: More detailed exploration of why participants receiving air experienced benefits similar to those receiving oxygen, and a fuller discussion of placebo, airflow, and psychological mechanisms underlying perceived benefit. The way it was well described on the discussion part "A recent scoping review showed that directing a stream of cool air to the face and nose may relieve breathlessness by stimulating the trigeminal nerve, leading to reduced inspiratory drive and modulation of neuronal activity within brain regions." Does it mean even Oxygen delivery have more placebo effect to alleviate from anxiety or

true breathlessness?

Thank you for these positive comments and for highlighting this important area. We agree that the similar perceived benefits reported by participants receiving oxygen and air require consideration of several possible mechanisms.
Additional airflow to the nose may influence the sensory perception of breathlessness, while using the device may also provide reassurance, reduce anxiety and increase confidence or willingness to undertake activity. Contextual and expectancy effects may therefore have contributed to experiences in both groups. However, these findings should not be interpreted as indicating that oxygen itself acts only through a placebo effect.
As SpO_2 was not measured during individual episodes of POC use, this qualitative study cannot distinguish physiological effects of oxygen from airflow, psychological or behavioural mechanisms. We have clarified this interpretation in the Discussion (Page 14) as follows:

> *" Using the POC may have increased confidence and willingness to undertake activity, regardless of the gas delivered. Contextual and expectancy effects may therefore have influenced both groups. However, similar experiences should not be interpreted as evidence that oxygen acted only through a placebo effect, as it may also have provided physiological benefit."*

One area that could be explored further is whether use or none-use of the POC (oxygen or air) may have caused unintended harms beyond the practical inconveniences described.

Thank you for raising this important point. We agree that potential unintended harms extended beyond practical inconvenience. Participants described physical effects, including nasal irritation, shoulder or back discomfort and interference with other equipment such as hearing aids, as well as psychosocial effects including embarrassment, self-consciousness and reluctance to use the POC in public. Some participants also reported greater breathlessness or less confidence when they were not using the POC.
We have clarified these points in the Discussion as follows:

> *" Potential unintended harms extended beyond physical discomfort and psychosocial effects may have discouraged POC use in public, however, the interviews did not assess whether non-use resulted in physiological or clinical harm."*

The study makes a valuable contribution to understanding patient experiences with ambulatory oxygen therapy in fibrotic interstitial lung disease and is suitable for publication after minor revision.

Thank you for your comment.

Reviewer: 4
Dr. Michael Hughes, Hampshire Hospitals NHS Foundation Trust
Comments to the Author:
Overall, it is a well-structured and logically presented qualitative study showing good real-world data and patient experience. The paper clearly describes the link to the previously published PFOX study and sets out logically the findings in a thematic approach. The examples chosen in the most part illustrate the overall thematic findings and the discussion place the findings in the wider conversation around the benefits of AOT.
The paper acknowledges the lack of transferability and the study design is open to bias, but allows deeper questioning.

Thank you for these positive comments and for recognising the value of the study design, thematic presentation and real-world patient perspectives. We appreciate the reviewer's acknowledgement of the strengths and limitations of the qualitative approach.

There are some aspects that are still unclear and impact the quality of the analysis:

1. How was adherence monitored/estimated? The perceived benefits may be significantly impacted by the overall adherence to therapy, but there is no comment if this was measured or how.

Thank you for highlighting this point.
We added information regarding device-recorded use to the Methods/Results as follows:

Methods (Page 4):

> *"The hours of POC use were recorded automatically by the device throughout the intervention period."*

Results (Page 7)

> *"POC use was low and varied considerably between participants included in this study with an overall mean use of 0.59 hours per day. At six months, mean daily use was 0.62 hours/day in the oxygen group and 0.57 hours/day in the air group."*

We have expanded the Discussion to acknowledge that the relatively low overall use of the POC may have limited participants' exposure to the intervention and influenced the benefits and burdens reported during the interviews.

Discussion page 17:

> *"The relatively low POC use observed in the parent trial should also be considered when interpreting participants' experiences.[9] Limited exposure to the device may have reduced opportunities to experience both potential benefits and burdens of POC use. However, the qualitative findings also provide insight into factors that may themselves have contributed to low use, including the practical limitations of the equipment and participants' individual decisions about when the perceived benefit justified the burden of using the device."*

2. There is no reference to any objective SpO2 improvement with POC O2 delivery (i.e. is the device at least showing a physiological improvement)? There is a potential ceiling effect of POC devices and thus the conclusions and comparisons would be more robust if there was evidence it did have a meaningful physiological impact that does or does not translates to a poor real-world improvement in QoL/confidence/exercise tollerance.

Thank you for raising this important point. SpO_2 was not measured during individual episodes of POC use, so we cannot determine whether perceived benefits were accompanied by an objective improvement in oxygenation. In the parent trial, the POC was used at its maximum pulse-dose setting, which may not have fully corrected exertional hypoxaemia in all participants. Previous work comparing POCs with compressed oxygen cylinders in ILD found similar oxygenation between devices, although substantial desaturation remained with both systems (PMID: 28544460). We have acknowledged this limitation in the Discussion (Page 18) as follows:

> *"The SpO_2 was not measured during individual episodes of POC use, therefore, perceived benefits could not be directly linked to an objective physiological response".*

3. Not all patients get on with a pulse-dose delivery style but seems to be little comment on this aspect.

Thank you for raising this point. The POC used in the trial delivered oxygen or air using a pulse-dose delivery mode at the maximum device setting. The interview guide did not specifically explore participants' preferences for pulse-dose versus continuous-flow delivery, or whether the device consistently triggered with inhalation. We therefore cannot determine the overall acceptability of the pulse-dose delivery mode from these data. However, one participant described the repeated pulsed sensation in the nostrils as distracting and uncomfortable. (P12, air group). We have clarified the delivery mode in the Methods, included this experience in the Results (Section 3.4), and acknowledged that acceptability of pulse-dose delivery was not systematically assessed as follows:

Methods (Page 5)

> *"Participants were provided with standard nasal cannulae and a standard POC carry bag, worn over one shoulder, but had the option to use a backpack if required or available. The POC provided pulse-dose delivery and was used at its maximal flow setting (Setting 5)."*

Results (Page 14)

> *"It was hard to concentrate on what you were doing because you had this constant sort of puff, puff in your nostrils and out of the accumulator on your back." (P12, air group)."*

Discussion (Page 18)

> *"The interviews did not specifically explore tolerance of pulse-dose delivery, preference for continuous flow or difficulties with breath-triggered delivery."*

4.Can authors confirm high-flow canulae were used with the device?

The Inogen One G3 POC is not a high flow device, so standard nasal cannulae were used. This has been clarified in the Methods (Page 5) as follows:

> *"Participants were provided with standard nasal cannulae and a standard POC carry bag, worn over one shoulder, but had the option to use a backpack if required or available."*

Specific comments on aspects of the manuscript:

Ref 11 in methods seems out of place (p8) as Nowell et al. (2017) does not seem to specifically link to the development of the coding tree and themes. On further review, reference 11 and 12 may be in the incorrect order as page 5 lists " Braun and Clarke (2006) and Nowell et al. (2017). as ref 11 and 12 respectively. Check please.

Thank you for identifying this issue. References 11 and 12 were listed in the incorrect order and have now been corrected, with Braun and Clarke (2006) cited as reference 11 and Nowell et al. (2017) as reference 12. We agree that Nowell et al. does not specifically support the development of the coding tree. We have also removed the Nowell et al. citation from the sentence describing development of the coding tree as follows:

> *Methods (Page 6):*
> *"Inductive, reflexive thematic analysis were conducted independently by two researchers (MH, CSW), guided by the interview guide and following the approach described by Braun and Clarke (2006)[11] and Nowell et al. (2017)[12]."*
> *"A coding tree was developed upon agreement between the researchers on themes and subthemes.[11]"*

It is unclear how the 23 invited participants from the 116 enrolled in the main study were

selected. On page 6, it is reported that "all participants were invited to take part" but then on page 9 it says of the 116 total, 23 were invited and all consented to take part in the study. Please clarify how the 23 were selected - or make clear that only 23 made it to the 6-month follow-up and thus 23 represents all participants.

Thank you for your comment. The qualitative study was conducted at three Melbourne trial sites, and all participants completing their six-month follow-up at these sites during the qualitative recruitment period were invited to participate. We have revised Results (Page 7) to clarify the recruitment process and removed the inaccurate reference to purposive sampling as follows:

> *"Of the 116 participants enrolled in the parent trial, 23 completed their six-month follow-up at one of the three Melbourne sites during the qualitative recruitment period and agreed participate."*

Table 2 should also include Interstitial Pulmonary Fibrosis in the legend.

Thank you for your comment. We have included the definition of IPF as idiopathic pulmonary fibrosis in the legend of Table 2

The overall thematic analysis would be strengthened if the total number of participants coded within each section from the O2 and air group were shown as well as the example quotes. i.e. "Participants from both groups frequently reported being less breathless, quicker recovery, and improved management of physical exertion". (Overall O2 n=4; air n=2) (p11). It would allow for a better comparison between the two groups for each theme, rather than just the examples given which can easily be biased from the examples chosen.
In addition, the above point would also benefit when "participants" is used but only one example given - i.e. 3.2 (p14). Clarify the total number of participants supporting this finding.

Thank you for this suggestion.
Braun and Clarke (doi:10.1191/1478088706qp063oa) explain that in thematic analysis, the importance of a theme is not necessarily determined by its frequency, but by whether it captures a meaningful pattern relevant to the research question. They further note that a greater number of instances or quotes does not necessarily make a theme more crucial to the research question.
Jowsey et al.(doi:10.1016/j.bjae.2021.07.006) similarly, state that thematic analysis does not rely on frequency of counts to indicate importance of the coded data.
Neale et al.(doi:10.1111/add.12408) acknowledge that numerical or semi-quantitative information may be problematic as the numbers could detract from the valuable nuanced and detailed data that is provided in a qualitative study and may lead readers to make inappropriate generalisations.
In line with the cited references, we agree that it is important to demonstrate that the findings are grounded in the experiences of participants from both group, however, reporting participant counts separately for the oxygen and air groups could imply a quantitative comparison of the prevalence of experiences, which this qualitative study was not designed to undertake. We have therefore retained the thematic presentation of the findings without adding numerical participant counts.

It is slightly unclear what the comparison in theme 3 is made to - in the study design these patients are AOT neive, so how can the participants notice an advantage of POC over any other method of O2 delivery? (p13-14). Please clarify what prior experiences allow patients to make this comparison, and to what?

Thank you for highlighting this lack of clarity. Although participants were not using ambulatory oxygen therapy at trial enrolment, one participant reported also having

experience using an oxygen cylinder and explicitly compared its finite supply with the ongoing oxygen supply generated by the POC. We have revised this section to clarify that this comparison reflected the experience of one participant rather than the participant group more broadly (Results - Section 3.2) as follows:

> *One participant who also reported having used an oxygen cylinder before appreciated the unlimited oxygen source generated by the POC, with the device able to operate from a power source and its batteries able to be., especially compared to cylinders.*
> *"The good thing about the portable is the continuous supply, can be 24 hours as long as you plug in the power source, there's no limit. But with the cylinder, if I finish 2 litres of that I finish 2 litres of that, I have to wait for new supply." (P4, O2 group)*

p14 section 3.3 use of "air group" rather than just "air" - consistency. Correct any other instances.

Thank you for noting this inconsistency. We have reviewed the manuscript and standardised the group descriptions accompanying all participant quotations to "oxygen group" and "air group" throughout.

The discussion draws on the key findings of the study and contextualises them in the current knowledge base. The authors identify the need of shared decision making because of the mixed benefits perceived (p18), but little insight if any of the data collected helps predict which patients will or will not get a benefit - indeed if there is any evidence to help in this decision. The discussion would be further strengthened if a comment could be made on this aspect.

Thank you for this important comment. This qualitative study was designed to explore experiences of POC use, and was not designed or powered to identify demographic, diagnostic or physiological predictors of treatment response. It is worthy of comment that there are currently no known quantitative predictors of benefit for ambulatory oxygen use in pulmonary fibrosis, such that a shared decision-making approach may be necessary to achieve the best outcomes for an individual patient. The discussion (Page 16) has been modified as follows:

> *"There are currently no data to suggest that demographic or physiological characteristics can distinguish people who benefit from AOT from those who do not. Because exertional breathlessness involves cognitive, sensory and emotional dimensions, its experience is inherently subjective. Understanding the role of AOT and POC therefore requires a phenomenological approach that accounts for participants' lived experiences and the personal meaning of their symptoms.[22] In the absence of established predictors of benefit, an individual trial of AOT with review of symptom response, activity goals and device burden may support shared decision making."*

The reference base is self-citation heavy. Whilst lots of relevant work has been published by this group, it would also be good to acknowledge briefly the wider base (i.e. studies also looking at POC devices and acceptability to patients, and different disease areas). I am also unclear of the difference between reference 9 and 13.

Thank you for noticing this. Reference 13 has now been removed, and the manuscript consistently cites reference 9.
We agree that the wider literature on patients' experiences of portable oxygen equipment should be more clearly represented so we have revised the Discussion to include evidence from other studies. (Page 16- references 24 and 25)

Please note also that the SRQR checklist is not cited within the main article as outlined in

section 2 of the checklist (which is correctly completed and included as a separate file). Please follow the guidance in the document.

Thank you for highlighting this omission. We have now cited the Standards for Reporting Qualitative Research (SRQR) guideline within the Methods section of the manuscript and added the corresponding reference to the reference list. The completed SRQR checklist remains included as a separate supplementary file.

VERSION 2 - REVIEW

**Reviewer**	**2**
**Name**	**Costello, Richard**
**Affiliation**	**Royal College of Surgeons Ireland, Respiratory Medicine**
**Date**	**19-Aug-2026**
**COI**	

---

thank you, my comments have been addressed

**Reviewer**	**4**
**Name**	**Hughes, Michael**
**Affiliation**	**Hampshire Hospitals NHS Foundation Trust**
**Date**	**22-Aug-2026**
**COI**	

Your replies to the comments by all reviewers are well thought through and rebuttals made with good rationale. Corrections have been made as appropriate and thus should be accepted for publication.
